# Determinants of Solid Fuel Use and Emission Risks among Households: Insights from Limpopo, South Africa

**DOI:** 10.3390/toxics10020067

**Published:** 2022-02-04

**Authors:** Rebecca O. Adeeyo, Joshua N. Edokpayi, Tom E. Volenzo, John O. Odiyo, Stuart J. Piketh

**Affiliations:** 1 Environmental Science Unit, Faculty of Science, Engineering and Agriculture, University of Venda, Private Bag X5050, Thohoyandou 0950, South Africa; 2 Department of Earth Sciences, Faculty of Science, Engineering and Agriculture, University of Venda, Private Bag X5050, Thohoyandou 0950, South Africa; Joshua.Edokpayi@univen.ac.za (J.N.E.); volenztom@gmail.com (T.E.V.); 3 Office of the DVC—Research, Innovation, Commercialisation, and Internationalisation (RICI), Vaal University of Technology, Vanderbijlpark 1900, South Africa; john.odiyo@univen.ac.za; 4 School of Geo and Spatial Sciences, North-West University, Potchefstroom 2520, South Africa; stuart.piketh@nwu.ac.za

**Keywords:** emissions, gaseous pollutants, household cooking, particulate matter, residential solid fuel, wood, possible health risk

## Abstract

Emissions from residential solid fuels reduce ambient air quality and cause indoor air pollution resulting in adverse human health. The traditional solid fuels used for cooking include coal, straws, dung, and wood, with the latter identified as the prevalent energy source in developing countries. Emissions from such fuel sources appear to be significant hazards and risk factors for asthma and other respiratory diseases. This study aimed at reporting factors influencing the choice of dominant solid fuel for cooking and determine the emission risk from such solid fuel in three villages of Phalaborwa, Limpopo province, South Africa. The study used descriptive analysis to show the relationship between the socio-economic variables and the choice of cooking fuel at the household level. Multiple correspondence analysis (MCA) was used further to detect and represent underlying structures in the choice of dominant fuels. MCA shows the diversity and existing relationship of how variables are related analytically and graphically. Generalised linear logistic weight estimation procedure (WLS) was also used to investigate the factors influencing choice of fuel used and the inherent emission risks. In the three villages, wood was the prevalent cooking fuel with 76.8% of participant households using it during the summer and winter seasons. Variables such as low monthly income, level of education, and system of burning are revealed as strong predictors of wood fuel usage. Moreover, income, water heating energy, types of wood, and number of cooking hours are significant (*p* ≤ 0.05) in influencing emission from wood fuel in the community. A notable conclusion is that variables such as income, education status and system of burning are determinants of wood fuel usage in the three villages, while income, water heating energy, types of wood and number of hours influence vulnerability to household emission and possible health risks in the use of solid energy sources.

## 1. Introduction

Since the 1952 classic episode of London smog with a record of about 4000 deaths within five days, air pollution has received great attention [1,2]. Air pollution has posed a negative influence on human health and the natural environment. It remains a growing problem, both in outdoor and indoor environments across the globe. In most developing nations, household solid fuel is a major cause of indoor air pollution. Globally, over three billion people are heavily dependent on residential solid fuels such as firewood, coal, crop residues and animal dung for their cooking energy needs, causing domestic air pollution. This results in about 2.5 million deaths annually [3,4,5]. The negative contributions of household air pollutions in Asian and African countries (disabilities adjusted life years) between 1990 and 2017 have been reported [6]. Great variation exists in the type of fuel used by low-income communities across developing countries and depends on the availability and affordability of resources.

Different appliances ranging from traditional stoves to improved cooking techniques are used in developing countries. Such stoves include the three-stone stove used in different parts of Africa, the Jiko stove commonly used in Kenya, the Pulumusa stove used in Zambia, the Tsotso stove used in Zimbabwe, the Justa stove in Honduras, the Eccoina stove in Guatamela, the single mud stove in India, and the Kang stove in China [7,8]. The energy ladder model was established to explain the transition process of residential fuel choices in developing countries [9]. The theory states that households will only move up the ladder from old-style to modern-style energy sources as their income level increases. Numerous residents of the low-income community cannot afford clean energy, thus, opt for traditional energy sources for domestic use [10,11,12]. In Asian countries, low-grade, fuel sources including wood, agricultural waste, cattle dung, and low-quality coal are used in rural and peri-urban areas. The use of three-stone stoves or simple open clay cookstoves is prevalent in rural parts of Asian countries. These stoves produce high products of incomplete combustion per unit of energy because of their low efficiency [13,14,15]. Additionally, about 600 million people living in the African continent have no access to electricity and rely on traditional forms of energy sources. In 2018, Africa Energy Outlook reported 490,000 premature deaths attributable to household air pollution in sub-Saharan Africa [5]. The fuels mostly used in this part of Africa are wood, charcoal, and agricultural residue, which influence the organic carbon and black carbon in the emitted particles [16]. However, wood remains the ubiquitous energy source for cooking and heating in many households of countries like Malawi, Zimbabwe, Ghana, Nigeria, Gabon, Angola, and South Africa [17].

**Figure 1:**
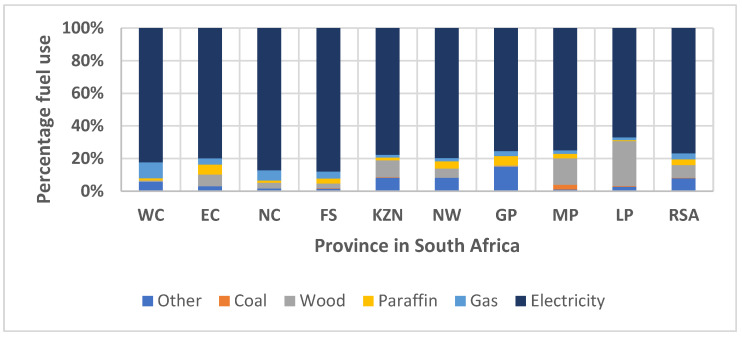
Percentage distribution of residential fuel for cooking in the Provinces of South Africa [28]. WC = Western Cape; EC = Eastern Cape; NC = Northern Cape; FS = Free State; NW = Northwest; GP = Gauteng; MP = Mpumalanga; LP = Limpopo; RSA = Republic of South Africa.

In South Africa, though more than 86% of households have physical access to electricity, low-income households and those without electricity still use diverse traditional solid fuels [18]. According to the Department of Energy (DoE), up to 35% of electrified households use other fuels, with 27% depending on solid fuels as the main source of energy for cooking [19]. Wood and coal are most prevalent, with coal being common around coal mines [20]. The percentage distribution of solid and liquid fuels used for cooking during 2018 in South Africa revealed wood fuel as prevalent in most of the provinces, except Gauteng and Western Cape (Figure 1). The appliance mostly used in the rural parts of South Africa are braziers commonly known as imbaulas [21]. In Limpopo Province, the use of three stone fire is dominant despite high electrification [22]. It was found that high concentrations of different pollutants are emitted from these solid fuels. These are much higher than the daily WHO air quality guideline (AQG) [23]. Diverse levels of particulate and gaseous pollution are associated with different solid fuels used in stoves within poorly ventilated kitchens [24]. Incomplete combustion of solid fuels releases large amounts of harmful pollutants, such as respirable and fine particulate matter (PM), as well as other gaseous substances [23]. Table 1 describes reported cases of pollutants in relation to residential wood burning in South Africa. Furthermore, Borrego et al. [25] explained different factors influencing the emissions from residential wood fuel, such as the type of wood, the structure of the fireplace or furnace, and showed that particulate matter emissions are significantly higher from a wood stove or a fireplace. Van den Berg [26] also reported domestic solid fuel use as a key source of particulate matter ambient concentrations, contributing up to 59.89% of PM_10_ and 58.67% of PM_2.5_. Research on residential fuel choice and the determinant factors is significant to understand the design and implementation of policymaking and energy transition [27]. 

Therefore, this study investigates factors influencing the choice of solid fuel usage and emissions risk in three selected informal settlements of Limpopo Province, South Africa, with a view to inform policymakers on policy interventions that address health risks associated with such choices and emissions.

## 2. Materials and Methods

### 2.1. Study Area

This study was conducted in Phalaborwa. The sites named Lulekane, Majeje and Makushane are villages in the neighborhoods of Phalaborwa, Ba-Phalaborwa Local Municipality, Mopani District, located in Limpopo Province and situated in the north-eastern part of South Africa. The choice of these sites was explained by their demographic characteristics. The climate of Phalaborwa is characterised by hot, wet summers and mild, dry winters, having a mean summer temperature of 24 °C, a mean winter temperature of 18.5 °C, and a mean annual rainfall of 481 mm [34]. The key energy resources in Limpopo Province are firewood, electricity, and LPG (liquefied petroleum gas).

### 2.2. Sampling and Data Collection

Qualitative questionnaires were distributed randomly to test the prevailing fuel type and identify demographic factors, kitchen characteristics, types of cookstove used, and other factors that influence emissions. The questionnaires were pretested in a similar community and the errors were adjusted before starting the real household survey. The sample size was determined using Rao soft sample size calculator with an acceptable error of 5% and was increased by 10% to get a total of 411 questionnaires. The number of questionnaires for each village was estimated using their population and a total of 371 participants responded with 133, 124 and 114 questionnaires from Lulekani, Makhusane, and Majeje villages correspondingly. Interviewers were engaged and trained to administer the questionnaire through a one-on-one interviewer-administered method in the local language (Xitsonga) of the villages. The data were collected using stratified random sampling. The data collected were analysed using cross tabulation in IBM SPSS Statistic 27 and the results were presented in bar charts. Ethical clearance (SES/20/ERM/06/1412) for this study was granted by the University of Venda and the Research Ethics Committee before conducting the study.

### 2.3. Multiple Correspondence Analysis (MCA)

Further statistical analysis was performed using multiple correspondence analysis (multivariate technique) to test the relationship that exists between the variables, using IBM^R^ SPSS^R^ statistics version 27.0 (SPSS Inc., Chicago, IL, USA). MCA allows relationships between both row and column variables, as well as between diverse levels of the individual variable, to be examined [35]. MCA is mainly useful when analysing more than two categorical variables. MCA is used to detect and represent underlying structures in a dataset in a low-dimensional Euclidean space. Therefore, it is unique in describing the patterns geometrically by locating each variable as a point in a low-dimensional space and the variables distributed along the dimensions. The closer the distance between points represented in space, the more similar the categories become in distribution [36]. However, MCA is considered as an exploratory tool for a dataset; it can be a very useful technique that helps to reveal groupings of variable categories in the dimensional spaces, providing key insight into relationships between them. The MCA method aids the measurement of significant contributing factors and degrees of association between factors through the analysis of the systematic patterns of variation within categorical datasets [37].

### 2.4. Generalised Linear Logistic Parameter Estimates

Statistical analysis was performed using generalised linear logistic weight estimation procedure in IBM^R^ SPSS^R^ statistics version 27.0 (SPSS Inc., Chicago, IL, USA) to investigate variables influencing emission among the households using wood fuel. Weight estimation procedure computes the coefficients of a linear regression model using weighted least squares (WLS), to give greater weight to predictors with less variability in determining the regression coefficients [38,39]. WLS tests a range of weight transformations that best fit the data. The weights can be interpreted as a change in the logarithm of the odds ratio E(β), associated with a one-unit change in any predictor (Equation (1)). Negative E(β) suggests decreasing the likelihood of occurrence as you increase the predictor variable, while positive E(β) indicates increasing likelihood occurrence as you increase the predictor variable. An odds ratio less than one implies that the variable decreases the likelihood of adoption whereas an odds ratio greater than 1 means that the variable increases the likelihood of adoption.

Ω = ez/(1 + ez)(1)

Ω is the probability of the event.
e is the base of the natural logarithms (about 2.718).
z is the linear combination and expressed as:
z = a + β_1_x_1_ + β_2_x_2_ + β_3_x_3_ … + β_i_x_i_
a is a constant (intercept).
βs = log odds coefficients estimated from the data.
x_s_ = values are the predictors the log of the odds ratio E(β); z = log (p/(1 − p));
P = probability of occurrence; and
1− p = probability of non-occurrence

## 3. Results and Discussion

### 3.1. Statistical Analysis of the Studied Villages

The demographic characteristics of the villages such as the education status, number of people in the household, and levels of income, as well as their type of kitchen, are presented in Table 2. In the three villages, it was revealed that wood was the prevalent type of cooking fuel by means of three stones. A total of 76.8% of participants use wood during the summer and winter seasons.

**Figure 2:**
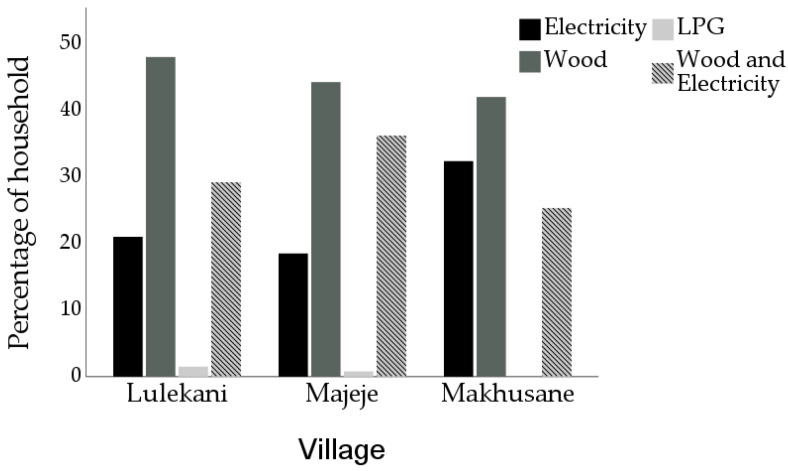
Percentage distribution of different cooking fuels during dry and wet seasons in the villages of the study area.

Figure 2 shows the percentage distribution of the cooking fuels used in the villages. Results revealed that 48.1%, 44.4%, 42.1% of the respondents use wood as their only fuel for cooking in Lulekani, Majeje and Makhusane, respectively. However, those who combine wood and electricity are 29.3%, 36.5% and 25.4% for Lulekani, Majeje, and Makhusane, respectively. Household participants using clean energy sources (electricity and liquid petroleum gas) in the three villages were less than 33%. Other biomass fuels are not used in these areas. The participant revealed their reasons for using wood as affordability and easy accessibility. Most times, the wood fuel is collected from a nearby bush by many of the participants, which are similar to other studies in sub-Saharan Africa [16].

**Figure 3:**
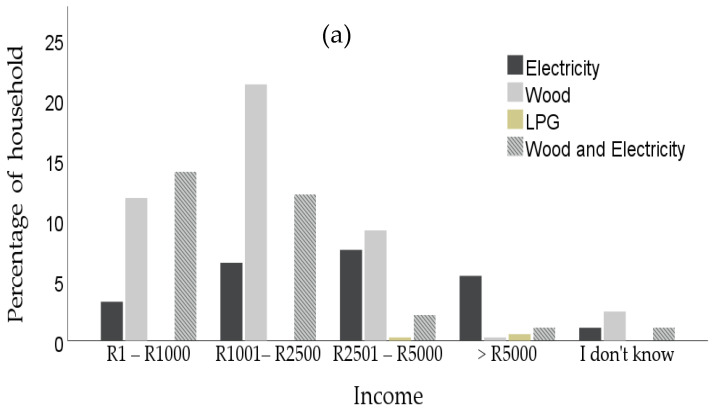

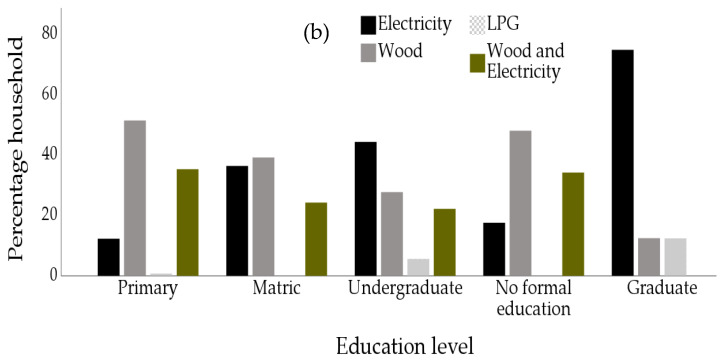
Percentage distribution of monthly income influencing the prevalence of cooking fuel (**a**) and education level influencing the prevalence of cooking fuel (**b**).

The results in Figure 3a revealed that the monthly income household has a strong relationship with wood fuel consumption in the three villages. Alem et al. [40] established that the demand for firewood for cooking is associated with the low income of the household. Indeed, 39.89% of participants households have an income between R1000–R2500 (US $69.49–US $172.88) with 21.29% using wood only, while 29.1% of participants households have an income less than R1000 (US $69.49), among which 11.9% are using wood only. It is observed that most of the respondents are females with low income, having their monthly income from social grant by the government, so their income depends on the number of their children, while some with income higher than R1000 (US $69.49) have their job as shopping mall attendants and petty traders. 

A Fisher’s exact test was administered to determine the relationship between monthly income earned and energy type, and has a value of 88.005, *p* > 0.000. This indicates that income earned, and energy type are significantly correlated, while the Cramer’s V symmetrical measure (0.299, *p* > 0.000) shows a moderate relationship between income and energy used. Rahut et al. [41] reported that the increase in income of the household increases the choice probability of clean cooking fuel over biomass, which is consistent with the results in this study. Additionally, the energy ladder theory claims that income is the most direct and vital factor in determining fuel choice. The theory found that a low-income household prefers to use old-style energy source such as firewood for cooking purposes. Other empirical studies revealed that an increase in income level results in the possibility that a household is likely to choose multiple fuel types concurrently instead of replacing firewood and other solid fuels use with a clean fuel, such as electricity and liquified petroleum gas (LPG) [42,43,44].Therefore, this study confirms the energy ladder hypothesis, which specifies that income has an important role in the demand for wood energy or cleaner energy for most households in Lulekane, Majeje and Makhusane.

Figure 3b presents the education status of the respondents in the three villages, of which they are mostly women. Results revealed that 35%, 29.1% and 28.8% have their education status as primary school, no formal education and matriculation status, among which 18.1%, 14.0% and 11.3% used wood only as their cooking fuel, respectively. Conversely, 4.96% and 2.2% have their education status as undergraduates and college graduates, among which those who use wood make up only 1.4% and 0.3%, respectively. This trend shows the low education status of the respondents from participant households. Other research has similarly revealed that low education level can influence the prevalence of firewood in some developing countries including Tajikistan, Cambodia, and Nigeria [45,46,47]. Moreover, Ouedraogo [47] and Rahut et al. [41] confirmed the influence of educational status in a household’s decision to adopt clean energy. It was therefore established that the higher the education level, the more the individual will opt for using clean energy sources, such as electricity or LPG. On the other hand, individuals with lower education levels may likely choose to use solid fuel, such as firewood. A higher education status may likely increase the income level, and thus, may increase the likelihood of choosing relatively cleaner fuels.

The Fisher’s exact test result has the value (59.604, *p* < 0.000). This shows that there is a significant correlation between the level of education and energy use, while the Cramer’s V symmetrical measure (0.231, *p* < 0.000) shows a moderate relationship between the level of education and energy use.

**Figure 4:**
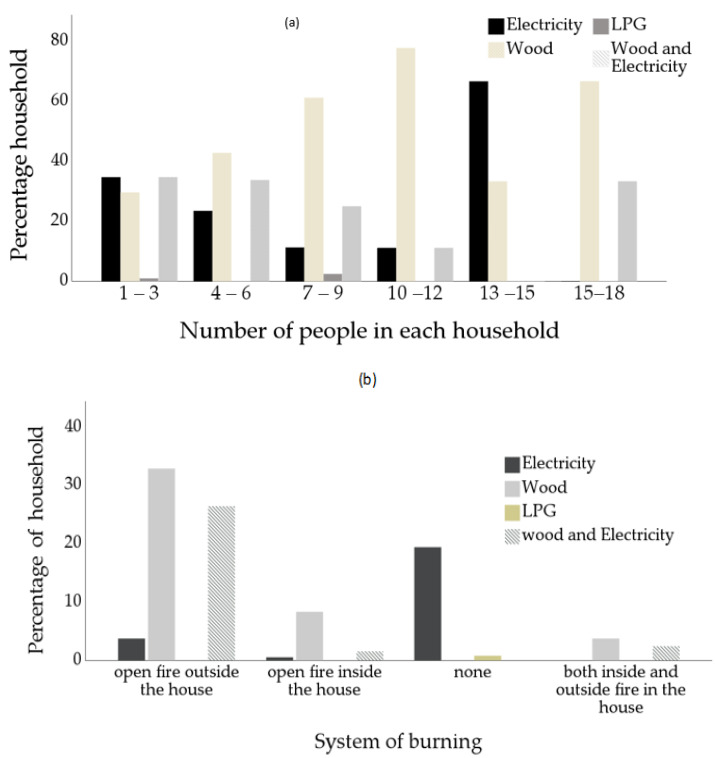
Percentage distribution of household size influencing the prevalence of cooking wood fuel (**a**) and the system of burning influencing the prevalence of cooking wood fuel (**b**).

The results in Figure 4a show that household size influences the type of fuel used in the three villages. Wood is the prevalent energy for cooking among the participants, with family sizes ranging from 4–6, 7–9, 10–12, and 15–18 consuming 42.8%, 61.3%, 77.8%, and 66.8% of wood, respectively. The average household size falls within the range of 4–6 (Table 2). This trend of wood use shows that an increase in household members is directly proportional to wood fuel usage. Moreover, some households with large family size explain that their decision to cook with firewood is due to its low cost. This would permit them to cook more food for the entire family, which may be expensive using clean fuels such as LPG or electricity. On the other hand, some members of small family size households explain that cooking with firewood may be due to availability of wood fuel, money-saving, and sometimes due to lack of electricity, as a result of load shedding from Eskom (Electricity Supply Commission). 

A Fisher’s exact test was used to determine the relationship between the variables having values 43.358, and *p* > 0.000. This shows a significant association between household size and firewood use in the villages, while Cramer’s V symmetrical measure of 0.194, *p* > 0.000 revealed a weak relationship between household size and the type of fuel used. This shows that household size may not necessarily influence the type of cooking fuel used in the three villages. However, Karakara et al. [48] stated that large household family sizes resulted in the possibility of using solid fuels such as firewood to meet their energy needs.

Figure 4b presents the system of burning influencing wood fuel usage in the three villages during winter and summer. Open fire outside house (OFOH) and open fire inside house (OFIH) using three-stone as the primary cooking stove is found among most participants’ households. Among those that use wood for cooking, 32.9% used open fire outside the house, 8.4% used open fire inside the house, while 3.8% used both inside and outside for cooking in the three villages. This shows that the two systems of burning exist in the villages among the wood fuel users. These burning systems may contribute to emissions both inside and outside the house; thus, exposure to indoor air pollution and equally ambient air pollution [49]. Bolling et al. [50] show from their studies that a mixture of soot and carbon emitted from open fireplaces contribute to pollution of the ambient air. 

Additionally, the result of the Fisher’s exact test revealed a value of 250.882, *p* > 0.000, which shows that the system of burning is significantly related to the energy used. At the same time, the Cramer V symmetrical measure was 0.495, *p* > 0.000, indicating a strong relationship between the system of burning and the type of energy used. This indicates both systems of burning influence the use of wood fuel.

### 3.2. Multiple Correspondence Analysis

The variables such as household size, types of cooking fuel, income, education level, and system of burning in the cross-tabulation above were studied in the MCA. From the MCA analysis, a two-dimension MCA solution was considered adequate. The first and second dimensions described are eigenvalues, 4.780 and 3.654; inertia, 0.531 and 0.406; and Cronbach’s alpha, 0.890 and 0.817. A reliable level for Cronbach’s alpha lies between 0.84 and 0.90 and a robust level is 0.81 in explanatory research as discussed by Taber, [51]. The values from this study showed homogeneity and strong interrelationship between the variables.

**Figure 5:**
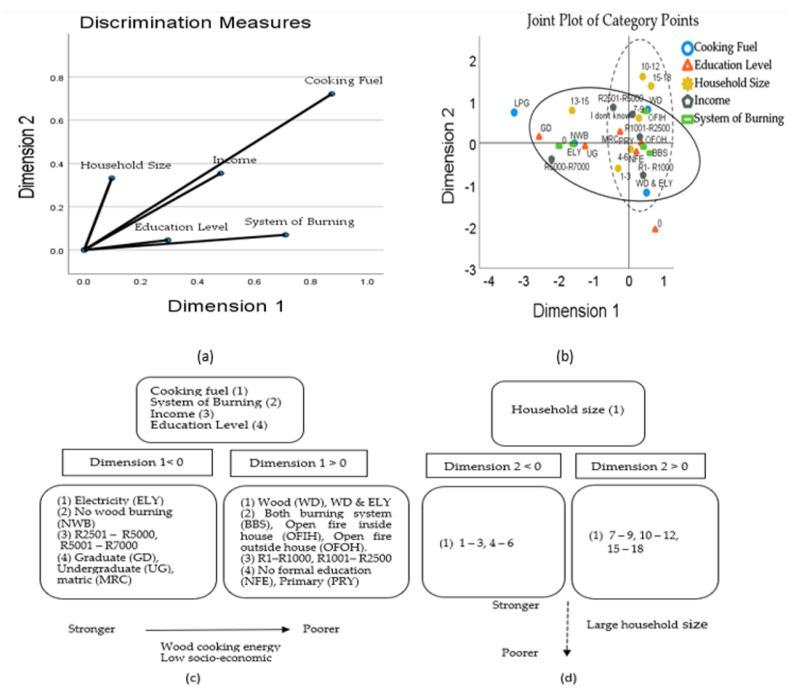
MCA dimensions discrimination measures (**a**), joint category plot of the explored variable categories (**b**), positive and negative centroid coordinates for dimension 1 (**c**), and positive and negative centroid for dimension 2 (**d**).

The MCA map in Figure 5 describes fuel and socio-economic variables in standard coordinates. MCA reduces the sum of squared distances between category points and respondents. For each of the variables, a discrimination measure, which is also known as a squared component loading, is calculated for each dimension. This measure is also the variance of quantified variables in that dimension. A maximum value of 1 is attained if the object scores fall into mutually exclusive groups and all object scores within a category are identical. Figure 5a,b represent the locations of variables that were identified by the MCA by mapping onto a two-dimensional graph. A clearer relationship among positive and negative centroid coordinates for both dimensions can be seen in Figure 5c,d. The variation explained by dimensions 1 and 2 was 53.11% and 40.59%, giving a total variance of 93.7 % (Table 3).

The variation categories with larger values contribute the most to the definitions of dimension. For cooking fuel (0.871), system of burning (0.710), income (0.481) and education level (0.295) are the most correlated for dimension 1. Likewise, the variable household size is correlated with dimension 2. In dimension 1, cooking fuel correlated (transformed variables) significantly with income (r= 0.458, *p* < 0.001), system of burning (r = 0.831, *p* < 0.001), and education level (r = 0.352, *p* < 0.001). Income correlated with system of burning (r = 0.376, *p*< 0.001), education level (r = 0.552, *p* = 0.001), and education correlated with system of burning (r = 0.353, *p* < 0.001). Similar correlations were found for dimension 2, except for household size which was not correlated. The correlations above 0.3 were considered only to have meaningful applied significance.

Overall, monthly income, respondent education status, and system of burning are the predictors of wood fuel use in the three villages. Findings from this study have shown that a statistically significant relationship exists between these variables and the type of fuel used for cooking. This is similar to findings in other sub-Saharan African countries by Makonese et al. [13], Mwaura et al. [52] and Menendez et al. [53], as well as other countries [54]. However, other socio-economic factors including age, sex, area of residence, cultural and behavioural factors (personal preferences, food taste, cooking speed, versatility), and other external factors can also influence the choice of household fuel [27,55]. The significance of each factor differs from location to location.

### 3.3. Generalised Linear Logistic Weight Estimation Procedure

Table 4 provides the weighted least squares. The negative E(βs) or odd ratios signifies the probability of reducing emission risks in terms of fuel adopted in the households, as the predictor variables—such as education level, vary wood categories, system of burning, quantity of wood bought, and number of hours of burning—increase.

Model predictors with positive E(β) or odds ratios signifies increasing probability of emission risks in terms of type of fuel used, as you increase the predictor variables such as wood use per day, sources of wood, income, household size, and number of households in the compound [56]. These predictors explained at least 88% of the variation in the model. However, only income (*p* ≤ 0.05), water heating energy (*p* ≤ 0.05), types of wood (*p* ≤ 0.05) and number of hours (*p* ≤ 0.05) are significant predictors that influence emission risk from fuel sources at the household level. The influence of income is probably through the amount of wood consumed. The effect of the type of wood could be explained in terms of its calorific value. However, the calorific value of the wood was not the subject of investigation. Studies established that emission from wood-burning varies and depends on diverse factors including combustion appliances, combustion conditions, the type of wood, and stages of the combustion cycle [50,57]. The size and composition of particulate matter, as well as gaseous emission, vary with diverse wood-burning appliances [58,59]. Findings have illustrated that traditional cookstoves led to more household air pollution compared to improved cookstoves [60]. Most populated countries like China and India reported high emissions of gaseous and particulate emissions from old-style wood stoves [26]. A recent survey also established that burning conditions influence the emission of particulate matter and gases due to physical factors of different wood species, such as the carbon content, which affects temperature as well as the density of the wood [61,62]. Guo et al. [63] studied the evaluation of particulate matter characteristics released from various wood species in subtropical China and revealed that combustion conditions of varying species of wood influence the contents. Besides, studies on the impact of wood species and burning conditions of particles emitted from residential wood stoves agreed that regardless of wood species, a proper behavioural operation is important to avoid unfavourable burning conditions [62]. Users can minimise the impact of fuel combustion during these operations through adequate awareness of burning conditions of the stove, regardless of different wood types [64]. Therefore, improving the behavioural and cultural style of the user is significant. Moreover, Volenzo and Odiyo [39] have found risk messaging (risk awareness) as critical in accounting for risk reduction behaviour. Thus, risk awareness could also be among missing the factors.

## 4. Potential Health Implications of Particulate and Gaseous Emissions from Solid Fuel

Epidemiological and toxicological studies provide overwhelming evidence that acute lower respiratory infections (ALRI), chronic obstructive pulmonary diseases (COPD) and lung cancer are among the major health risks from indoor air pollution associated with biomass-based fuel [65]. Other health risks include cataracts, tuberculosis, asthma attacks, and lower birth weight [66,67]. Emissions from residential solid fuel also worsen the suffering and shorten the life of people suffering from malaria, tuberculosis, HIV/AIDS, chronic cardiovascular diseases, and respiratory diseases. Research has linked long-term exposure to firewood smoke with reduced pulmonary functions, chronic bronchitis, heart problems, and premature mortality [68]. Short term exposure has been associated with acute bronchitis, asthma attacks, and greater vulnerability to respiratory infections, leading to high respiratory cases in hospital admissions.

Gioda et al. [69] observed higher respiratory symptoms in the population exposed to wood burning compared to individuals who used LPG. Cough and coryza were found to be the most prevalent symptoms in both adults and younger individuals. An increase in cancer has also been associated with indoor cooking with firewood [70]. Empirical studies have estimated the occurrence of cancer in the oral cavity (tongue and other parts of the mouth), upper aerodigestive tract, and oesophagus, to associate with exposure to firewood burning in Brazil [71,72]. Furthermore, Khan et al. [71] studied the effect of using wood fuel and showed that children in a household with such fuels are 1.5 times more likely to have symptoms of acute respiratory infections (ARI) than children using clean fuels. In a study conducted with indigenous Guarani, children under five years of age, belonging to 83 communities in Brazil, were found with hospitalisation for acute lower respiratory tract infection (ALRTI), which was associated with the use of firewood. An epidemiological report for South Africa indicated the highest peak of ill health among children below the age of five years and the elderly people [73]. Sanyal et al. [74] reported that a high level of recurring respiratory infections was experienced amongst children in the Eastern Cape Province of South Africa after being exposed to coal and firewood emissions. The World Health Organization reported that an estimate of about 1400 lives in South Africa were prematurely lost because of household air pollution between 2007 and 2012. Nearly half of this number includes children below five years and are affected by acute lower respiratory infections [75].

A significant association is also found between the use of wood as cooking fuel and asthma, and increased daytime respiratory symptoms. Additionally, empirical reports revealed a significant relation between wood use and respiratory outcomes among different genders in South Africa and other counties in sub-Saharan Africa [76,77,78]. A recent report from the University of Auckland presented many studies where asthma is found to be among those affected by wood smoke. Orozco-Levi et al. [79] also strongly associated exposure to wood smoke with chronic obstructive pulmonary disease (COPD). The length and intensity of exposure in both summer and winter were related to the risk of COPD in developing countries [80]. Thus, this shows that asthma and day-time respiratory symptoms could be associated with some of the residents of Majeje, Lulekane, and Makhusane villages.

## 5. Recommendations and Research Gaps

Generally, reduction in emissions from household wood fuel combustion is enormously important not only to improve health and air quality for lots of people in developing nations but also to alleviate regional and global climate change. Awareness is important to improve behavioural patterns of the user during the use of solid fuels. Awareness improves education status or knowledge level, which may contribute to an increase in income and high socio-economic status. Future studies could investigate the role of risk communication in the choice of energy type. Enforcement of local interventions that incorporate ventilation technology upgrading and national governmental policies should be pursued. Regardless of the knowledge on the health effect of wood and other solid fuels in developing countries, enough empirical evidence on household cooking wood and associated health effect is still lacking.

## 6. Conclusions

Wood is found to be the dominant fuel for cooking among the residential solid fuels used in rural parts of developing countries, with either traditional three-stone or improved wood stove technology. In the studied villages, low monthly income, level of education, and system of burning are influencing their choice of using wood fuel for cooking purposes, while household size may not be a contributing factor. Emissions from these wood fuels appear to be a significant possible hazard for asthma and other respiratory diseases among households and represented demographics. A notable conclusion is therefore established that variables such as education status, income, and system of burning are the predictors contributing to the use of traditional energy sources. In addition, income, water heating energy, types of wood and number of hours are vulnerable factors to emission risk from households and possible health risks during wood usage as energy sources.

## Figures and Tables

**Table 1:** Studies assessing emissions associated with residential wood burning in South Africa.

References	Study Design	Population	Sample Size	Exposure	Reported Pollutant Concentration
[29]	Case study	Kwadela. Mpumalanga, South Africa	One household	Monitoring of household in winter, 2013 and 2014, summer 2014 and 2015 for ambient air pollution of Pm_10_ and Pm_2.5_.	Mean PM_2.5_, and Pm_10_ are 27 ± 18 µg/m^3^ and 48 ± 122 µg/m^3^, respectively.
[30]	Cross-sectional study	Children (≤15 years of age) who participated as case controls in the TB study with eThekwini Municipality, Durban, KwaZulu Natal, South Africa	114 households	Environmental air sampling of indoor air pollutants associated with the combustion of cooking fuels and second hand smoke (SHS) was conducted in 114 of them.	Mean (range) indoor concentrations of PM_10_, NO_2_ and SO_2_ were 64 µg/^3^ (6.6–241.0); 19 µg/m^3^ (4.5–55.0) and 0.6 µg/m^3^ (0.005–3.4), respectively.
[31]	Cross-sectional study	Households of pregnant women in Durban (North and South). participants are the mother and child in the environment	300 households	Collection of information on household building, occupants, and outdoor sources, such as industries and major roads in the vicinity of the homes. Pm_2.5_ levels were measured in 300 homes for a period of 24 h.	The PM_2.5_ levels ranged from 1.4 to 162.0 µg/m^3^. The mean (SD) of these levels was 38.3 (31.1) µg/m^3^, and the median was 28.0 µg/m^3^.
[32]	Intervention study	Two poor rural villages in Mafikeng municipality, Northwest South Africa	219 households	Children living in outdoor-burning homes showed significantly lower (88–90%) levels of exposure to CO. Children experience high levels of indoor air pollution when fires are brought indoors compared to indoor-burning homes at both assessments.	The mean child exposure to CO by outdoor burning for baseline is 0.5 ppm and follow up is 0.3 ppm, while indoor burning for baseline is 4.2 ppm and follow up is 3 ppm.
[33]	Panel study	Kwadela, Mpumalanga, South Africa	20 households	Monitored over two years: two summers and two winters (10–12 weeks each); 207 household’s questionnaires were administered to determine household fuel use and supposed quality of life.	Solid fuel use: coal (75.36%) and wood (63.28%). 40.57% of households used a combination of these fuels. PM_10_ concentrations were 102.1 ± 76.96 and 99.29 ± 61.39 (µg/m^3^), respectively, and summer concentrations were 50.43 ± 29.59 and 66.03 ± 25.86 (µg/m^3^).

**Table 2:** Demographic characteristics of the case study villages in Limpopo Province.

Factors	Parameters	Lulekane *n*= 133 n (%)	Majeje *n*= 124 n (%)	Makhusane *n* = 114 n (%)
	No formal education	42 (31,6)	33 (26,6)	33 (29,0)
	Primary	53 (39,9)	54 (43,6)	23 (20,2)
Education Level	Matric	32 (24,1)	28 (22,6)	47 (41,2)
	Undergraduate	4 (3,0)	6 (4,8)	8 (7,02)
	Graduate	2 (1,5)	3 (2,4)	3 (2,6)
	1–3	32 (24,1)	28 (22,6)	38 (33,3)
	4–6	57(42,9)	60 (48,4)	49 (43)
No. people per	7–9	34 (25,6)	26 (21,0)	20 (17,4)
Household	10–12	7 (5,3)	8 (6,5)	3 (2,6)
	13–15	1 (0,8)	2 (1,6)	3 (2,6)
	16–18	2(1,5)	0(0)	3(2,6)
Income	<R1000	40(30,1)	47 (37,9)	21 (0,16)
	R1001–2500	52 (39,1)	41 (33,1)	55 (48,2)
	R2501–R5000	27 (20,3)	21 (16,9)	23 (20,2)
	>R5001	5 (3,8)	11 (8,9)	11 (9,65)
	I don’t know	9 (6,8)	4 (3,2)	4 (3,51)
	Open fire inside a kitchen	89 (66,9)	77 (62,1)	66 (57,9)
Type of Kitchen	Open fire outside the house	14 (10,5)	19 (15,3)	6 (5,3)
	Both inside and outside	6 (4,5)	10 (8,1)	5 (4,4)
	None	24 (18,1)	18 (14,1)	37 (32,5)

*n* = number of samples, n= frequency.

**Table 3:** Multiple correspondence analysis (MCA) dimension discrimination measures.

Varriables	MCA Dimension		Mean
	1	2	
Income	0.481	0.354	0.418
Education level	0.295	0.046	0.710
Cooking fuel	0.873	0.722	0.797
Household size	0.097	0.332	0.215
System of burning	0.710	0.070	0.390
Active total	4.780	3.654	4.217
% of variance	53.11	40.59	46.854

**Table 4:** Generalised linear logistic parameter estimates on fuel use and factors influencing emission risk in three villages of Phalaborwa Limpopo Province.

Unstandardized Coefficient			Standardized Coefficient			
Variables	E(β)	Std. Error	E(β)	Std. Error	t	Sig.
(Constant)	3.858	0.724			5.328	0.000
Education	−0.061	0.076	−0.036	0.045	−0.807	0.421
HH in compound	0.004	0.143	0.002	0.053	0.032	0.975
HH size	0.003	0.112	0.001	0.050	0.023	0.981
Income	0.271	0.096	0.152	0.054	2.823	0.006
Water heating Energy	0.456	0.064	0.470	0.066	7.174	0.000
Categories of wood	−0.002	0.079	−0.001	0.068	−0.021	0.983
Types of wood	−0.287	0.125	−0.228	0.099	−2.290	0.024
Sources of wood	0.133	0.141	0.058	0.062	0.943	0.347
Wood prices	−0.038	0.052	−0.044	0.062	−0.721	0.472
Quantity of wood bought	−0.056	0.143	−0.036	0.091	−0.391	0.697
Wood use per day	0.108	0.133	0.055	0.068	0.817	0.416
System of burning	−0.013	0.139	−0.007	0.068	−0.096	0.924
No. of burning hours	−0.281	0.083	−0.322	0.096	−3.365	0.001
No. of burning days/week	−0.093	0.064	−0.074	0.051	−1.463	0.146

Multiple R = 0.88; R^2^ = 0.774; Adjusted R^2^ = 0.746; Log-likelihood = −210.34; *p* = 0.00.

## Data Availability

Not applicable.
